# Ensifer-mediated transformation: an efficient non-Agrobacterium protocol for the genetic modification of rice

**DOI:** 10.1186/s40064-015-1369-9

**Published:** 2015-10-13

**Authors:** Evelyn Zuniga-Soto, Ewen Mullins, Beata Dedicova

**Affiliations:** International Center for Tropical Agriculture (CIAT) Transformation Platform, International Center for Tropical Agriculture (CIAT), A.A. 6713 Cali, Colombia; Department of Crop Science, Teagasc Crops Research Centre, Oak Park, Carlow, Ireland; Department of Plant Breeding, Swedish University of Agricultural Sciences (SLU), Box 101, Sundsvägen 10, 23053 Alnarp, Sweden

**Keywords:** Transformation, Rice (*Oryza sativa* L.), *Ensifer adhaerens* OV14, T-DNA integration

## Abstract

While *Agrobacterium*-mediated transformation (AMT) remains the most widely used technique for gene transfer in plants, interest exists for the use of non-*Agrobacterium* gene delivery systems due to freedom-to-operate issues that remain with AMT across several jurisdictions. In addition, the plant pathogenic mode of action of *Agrobacterium tumefaciens* significantly increases the costs to passage engineered cultivars through the regulatory process. *Ensifer adhaerens* (OV14) is a soil-related bacterium with the proven ability to genetically modify the model plant *A. thaliana* and the staple crop *S. tuberosum* (Wendt et al., Trans Res 21:567–578, 2012). While previous work was relevant for dicotyledonous species, in this study, the efficacy of *Ensifer adhaerens* (OV14)-mediated transformation (EMT) was determined on two japonica rice varieties, Curinga and Nipponbare, and the recalcitrant indica variety, IR64. The results indicated that *E. adhaerens* (OV14) exhibits infection efficiencies ranging between 50–70 %, 90–100 % and 90–95 % for Curinga, Nipponbare and IR64 respectively. Curinga and Nipponbare plants transformed with *E. adhaerens* (OV14) and *A. tumefaciens* (LBA4404 and EHA105) were regenerated achieving transformation efficiencies of 16 % and 26–32 % for Curinga and 7 and 4 % for Nipponbare respectively. Separately, the transformation of IR64 was only recorded via EMT (transformation efficiency ~1 %). Integration analyses conducted on 24 transgenic rice lines illustrated that T-DNA insertion occurred randomly throughout the rice genome for EMT (and AMT), with similar integration patterns in the rice genomic DNA observed for both bacterial species.

**Electronic supplementary material:**

The online version of this article (doi:10.1186/s40064-015-1369-9) contains supplementary material, which is available to authorized users.

## Background

Rice (*Oryza sativa* L.,) remains a stable food for more than half of the world’s population but entered the new millennium facing a series of significant challenges relevant to a/biotic stress. Despite the fact that its yields have doubled over the last three decades (FAOSTAT 2013) a constant need exists for new rice cultivars to be developed (Zhang 2007) be that through conventional breeding practises or genetic engineering platforms. While Agrobacterium mediated transformation (AMT) is widely adopted as the system of choice for engineering novel crop varieties freedom-to-operate issues remain (Chi-Ham et al. 2012), especially in regard to regional-specific licensing (e.g. in the USA due to US Patent No. 8273954). The cost of securing regulatory approval for AMT-derived lines is also significant primarily due to the plant pathogenic nature of *A. tumefaciens* (CropLife International 2011), which in turn ensures that bringing engineered crop lines through to commercialisation is typically an option exclusive to the largest ag-biotech companies.

Addressing this would require a gene delivery system that is based on a non-pathogenic organism with rates of transformation equivalent to that of AMT for both dicot and monocot species. In 2005 Broothaerts and collaborators demonstrated the efficiency of different *Rhizobia* species such as *Sinorhizobium meliloti*, *Mesorhizobium loti* and NGR 234 (collectively called Transbacter). A more recent approach to the use of non-*Agrobacterium* species for plant transformation was achieved in 2012 when Wendt et al. (2012), identified the bacterium *Ensifer adhaerens* strain OV14 as a member of the *Rhizobiaceae* family with the potential to successfully transform *A. thaliana* and *S. tuberosum*.

Similar to *A. tumefaciens, E. adhaerens* is a gram-negative bacterium belonging to the alphaproteobacteria class; while predatory of prokaryotic cells (Martin 2002) *E. adhaerens* appears to be a plant beneficial bacterium (Zhou et al. 2013). Phylogenetically, this organism is related to non-pathogenic bacteria belonging to the same genera (Garau et al. 2014; Reeve et al. 2014) and the genome sequencing of *E. adhaerens* clearly identified its separation from the *Agrobacterium* dominated clade of the *Rhizobiaceae* family (Rudder et al. 2014). In comparing the genome of *E. adhaerens* against *A. tumefaciens* C58 and *S. meliloti* 1021, the same study concluded that while the *E. adhaerens* and *S. meliloti* genomes possessed homologs to chromosomal based genes cited as essential to *A. tumefaciens* T-DNA transfer, it was the *E. adhaerens* genome that also included genes that while non-essential for transformation do exert a positive influence on virulence and the ability to genetically transform host tissues.

For the regulatory evaluation of engineered varieties, potential risks associated with the product released, including the erroneous presence of vector backbone sequences flanking the T-DNA (FAO 2004) need to be evaluated. It is already known that the expression level of introduced genes can vary substantially between individual transformed plants, probably as a consequence of the influence of different flanking DNA sequences and T-DNA integration patterns (Dietz-Pfeilstetter et al. 2003). For example, based on AMT (dependent on *Agrobacterium* strain and vectors used), between 20 and 80 % of transgenic plants may contain backbone sequence (Van der Graaff et al. 1996; Kononov et al. 1997; Hanson et al. 1999; De Buck et al. 2000). More specifically, in rice, separate studies have noted the presence of non-T-DNA sequences in 33 % (Yin and Wang 2000), 45 % (Vain et al. 2003), 38 % (Zhai et al. 2004) and indeed in up to 66 % of transgenic lines generated (Afolabi et al. 2004), which serves to complicate any pre-market regulatory assessment should it be required (Wilson et al. 2006). In the present study we describe the protocol development of *Ensifer adhaerens* (OV14)-mediated transformation for two Japonica rice varieties (Curinga and Nipponbare) and the commercially important indica variety IR64 by using the unitary transformation plasmid pCAMBIA 5105. The protocol applied for Curinga and Nipponbare (*Oryza sativa* L. spp. japonica) is based on the CIAT rice protocol developed for various japonica rice species (Tabares et al. 2011); and that for IR64 (*Oryza sativa* L. spp. indica) is based on Datta and Datta (2006) albeit with several modifications. Furthermore we evaluated the T-DNA integration patterns found in rice plants transformed via *E. adhaerens* (OV14) and *A. tumefaciens* (LBA44404 and EHA105 with the pCAMBIA 1305.2 plasmid) in order to evaluate (1) propensity for backbone sequence integration, and (2) insertion patterns (e.g., truncations, filler sequences, and duplications) within the post-integration genome.

## Results

### Comparative assessment of *Ensifer adhaerens*-mediated transformation and *Agrobacterium*-mediated transformation in rice

**Fig. 1:**
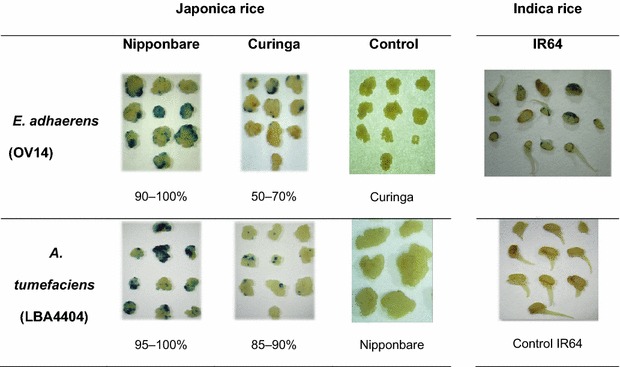
Infection efficiencies of *E. adhaerens* (OV14) and *A. tumefaciens* (LBA4404) in Curinga and Nipponbare japonica rice varieties, and of *E. adhaerens* (OV14) in the IR64 indica rice variety

The infection efficiency of *E. adhaerens* (OV14) was tested in embryogenic Curinga and Nipponbare calli and in immature embryos of IR64 using a histochemical GUS assay (Fig. 1). Comparing the two japonica varieties, it was found that var. Nipponbare was more amenable for infection with *E. adhaerens* (OV14), exhibiting transient GUS activity in more than 90 % of treated calli, meanwhile only 50–70 % of treated Curinga-derived calli reported to be positive for GUS staining following EMT. On the other hand, *A. tumefaciens* (LBA4404) infection of Nipponbare and Curinga induced GUS staining in between 95 and 100 % and 85–90 % of treated calli, respectively. The infection rate obtained for IR64 with *E. adhaerens* (OV14) was 90–95 %.

**Fig. 2:**
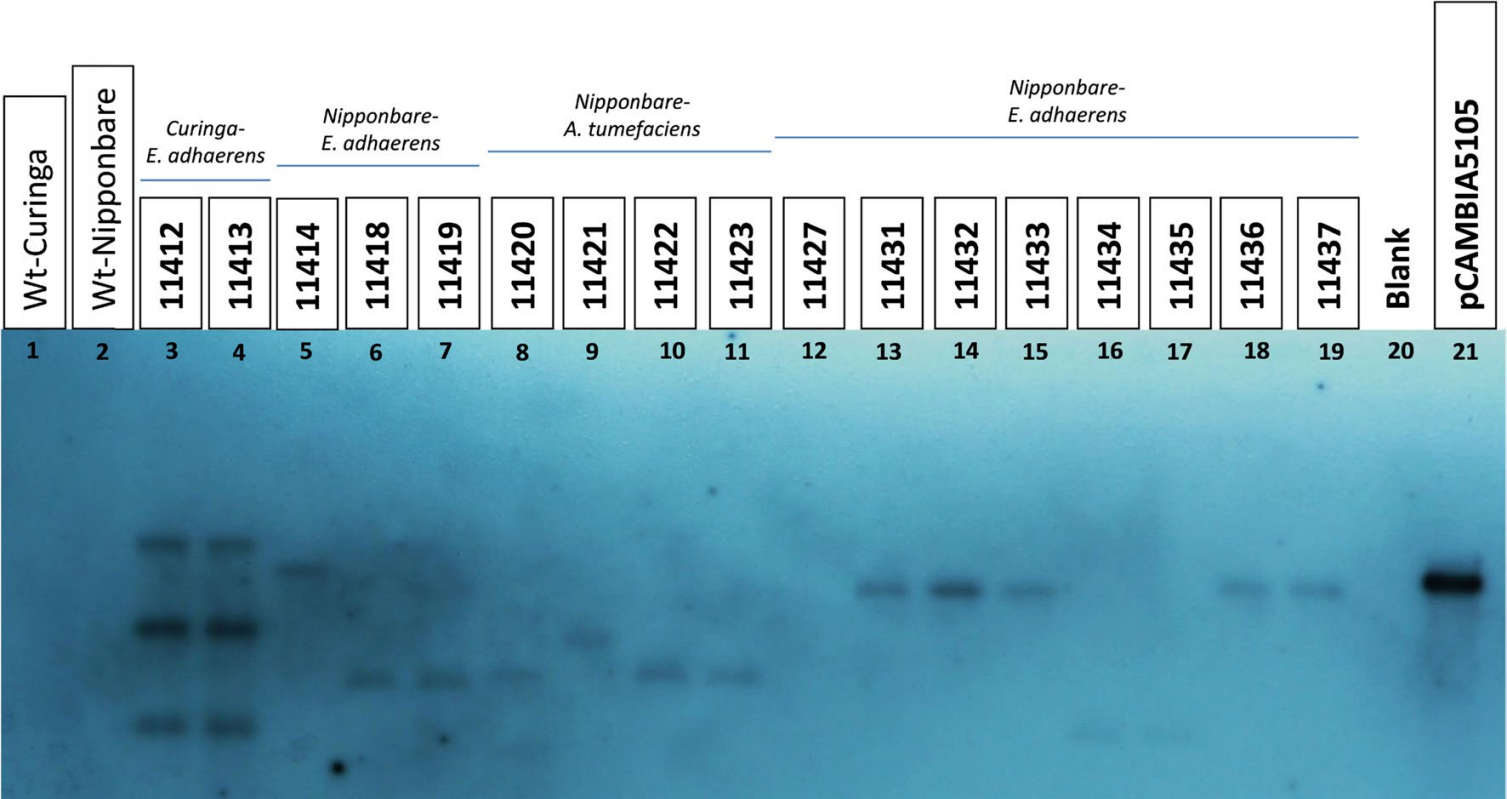
Southern analysis of transgenic rice plants using *hptII* probe. *Lanes 1 and 2* non transgenic control DNA from Curinga and Nipponbare respectively. *Lanes 3 and 4* T0 plants with multiple copy events. *Lanes 5*–*19* T0 plants with single copy events. *Lane 20* no sample. *Lane 21* Plasmid DNA from PCAMBIA 5105

From the EMT pipeline the number of transgenic plants regenerated from hygromycin-resistant calli varied among cultivars. For example, from 250 Nipponbare calli, 18 independent Southern blot positive plants were obtained, yielding a transformation efficiency of 7.2 %. Fifteen of these turned out to be single copy events, representing 83.3 % of the tested population. For Curinga, 275 calli delivered 45 Southern blot positive plants (transformation efficiency of 16.3 %), of which 56 % were deemed single copy events (Table 1; Fig. 2).

**Table 1:** Summary table of the transformation events obtained by infecting rice varieties with *A. tumefaciens* (EHA105 and LBA4404) and *E. adhaerens* (OV14)

Bacterial strain	Rice variety	No. of infected calli	Plants delivered	PCR (+)	Southern blot (+)	1C	2C	3C	Transformation efficiency (%)
*E. adhaerens*	Nipponbare	250	69	20	18	15	1	2	7.2
*E. adhaerens*	Curinga	275	73	54	45	25	14	6	16
*A. tumefaciens* (EHA105)	Curinga	25	14	11	8	7	1	0	32
*A. tumefaciens* (EHA105)	Nipponbare	100	4	4	4	4	0	0	4
*A. tumefaciens* (LBA4404)	Curinga	400	133	121	106	56	42	8	26.5
*E. adhaerens*	IR64	100	1	1	1	1	–	–	1

C stands for “copy number”

For *A. tumefaciens* (LBA4404-pCAMBIA 1305.2); from 400 Curinga infected embryogenic calli (collated from independent experiments) a transformation efficiency of 26.5 % was obtained as per independent integration pattern as per Southern blot. With *A. tumefaciens* (EHA105-pCAMBIA 1305.2), only a single experiment was carried out on Curinga (n = 25 embryogenic calli) and on Nipponbare (n = 100 embryogenic calli), which delivered infection efficiencies of 32 and 4 %, respectively. For IR64 infected with *E. adhaerens* (OV14), a transformation efficiency of 1 % was recorded from 100 immature embryos but using *A. tumefaciens* (EHA105-pCAMBIA 1305.2) no transgenic plants were obtained (data not shown).

### Analysis of T-DNA flanking sequences in transgenic plants

A chromosome walking approach was employed to characterise the presence/absence of backbone integration at the T-DNA integration sites of EMT and AMT regenerated plants. Twenty four and 13 flanking sequences were obtained for the right and left borders, respectively, from 22 independent single copy transformants of Curinga and Nipponbare using the bacterial strains *A. tumefaciens* (LBA4404 and EHA105) and *E. adhaerens* (OV14). The sample 11,197/11,199 corresponds to two “sister” plants originated from the same calli and was used as a control.

Of significance, none of the analysed sequences showed the erroneous integration of adjacent backbone DNA. Evaluating the adjacent sequences from the right border, two different types of configurations were found associated with the T-DNA insertion for both *E. adhaerens*- and *A. tumefaciens*-transformed plants: (1) partial deletion of the border, (2) complete deletion of the border and adjacent T-DNA.

In agreement with previous reports (Tinland 1996; Stahl et al. 2002; Kumar and Fladung 2002; Zhai et al. 2004) no nucleotides beyond the TGA right-border sequence were found for any line in the present study. Of the 16 japonica plants generated with EMT, four contained the three nucleotides, TGA (line 11,232, 11,422, 1197/99 and 11,473); one, the nucleotides TG (line 11,267); and four only the nucleotide T (line 11,379, 11,291, 11,414 and 11,471). In three of the sequences analysed, DNA integration led to the loss of >100-bp of T-DNA (line 11,309, 11,420 and 11,280). Lines with 7-bp (line 11,436 and 11,267), 11-bp (line 11,645) and 25-bp (line 11,253) corresponding to filler sequences were also found. Junctions in which the whole 25-bp of the right border was lost, were found in four lines (11,814, 11,436, 11,260 and 11,813). In addition, microhomologies of 4-bp (TGCA) were identified in lines 11,309, 11,420, 11,470, 11,418 and 11,267. A 25-bp fragment in the right border (adjacent to the T-DNA) known to correspond as part of the region 3′ UTR polyA signal from the *hpt II* gene was identified in line 11,253, this fragment is known to be originally located at the left border of the T-DNA. For the 12 EMT-derived Japonica right-border sequences evaluated, the right border was partially absent from six of them (line 11,197/99, 11,418, 11,473, 11,276, 11,414, 11,471), whereas five possessed partial deletions ranging from 17 to 200-bp (line 11,470, 11,436, 11,253, 11,694 and 11,280). Of the nine AMT-derived japonica sequences assessed, five of them lost the complete length of the right border (line 11,289, 11,309, 11,420, 11,645 and 11,814), while the other four had between one and three bases of the right border present (line 11,379, 11,291, 11,232 and 11,422).

Twelve left-border sequences were retrieved for both AMT and EMT regenerated plants. Similarly to the right border, none of the samples showed an integration of erroneous vector backbone sequence. For the left border, characteristics such as truncation of the border and the T-DNA along with filler sequences were noted. Of the six EMT generated plants that were analysed, four contained remaining left-border portions ranging between 10 to 19-bp (line 11,197/99, 11,260, 11,473 and 11,813) with sample 11,694 also containing a filler segment of 6-bp. One line (11,471) exhibited a truncation of 31-bp of the T-DNA. Among the five analysed plants obtained with *A. tumefaciens* (LBA4404 and EHA105), three contained left-border nucleotides ranging between 9 and 17-bp (line 11,291, 11,379 and 11,232), two lines (11,645 and 11,422) contained deletions of 100 and 277-bp respectively, also the line 11,422 included a filler segment corresponding to a 16-bp fragment of the *hpt II* gene (Additional file 1: Table S1).

### Deleted rice genomic sequences

T-DNA integration is often accompanied by a small deletion in the plant DNA at the site of insertion (Gheysen et al. 1990). In this study, we analysed a total of 11 transgenic independent lines, all of which showed different degrees of deletion of the rice genome. In the six EMT lines evaluated the deletions ranged between 16 to 273 bp and for the five AMT lines were between 11 to 176 bp (Additional file 1: Table S1).

### Integration sites in rice chromosomes

**Fig. 3:**
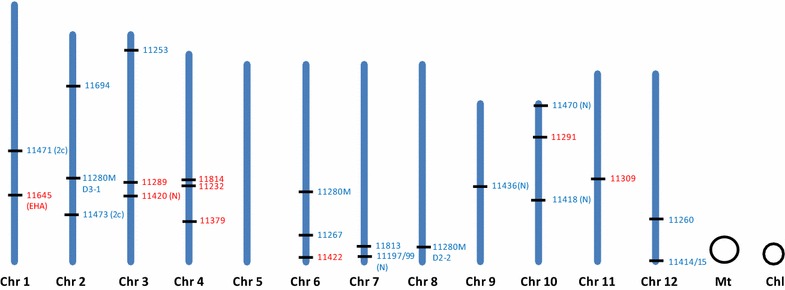
Map positions of T-DNA insertions derived from independent transgenic lines. The 12 rice chromosomes are displayed in* blue*; mitochondrial and chloroplast DNA are indicated as *black circles*. T-DNA insertions belonging to *E. adhaerens* and *A. tumefaciens* are indicated in *blue* and *red* respectively

Mapping the T-DNA flanking sequences on the rice chromosomes revealed no apparent chromosomal position preference. T-DNA insertions appeared to be randomly distributed for both *E. adhaerens* and *A. tumefaciens* (both strains) obtained events. Moreover, the insertions appeared to be evenly distributed along the chromosomes within the rice genome (Fig. 3).

For *E. adhaerens* (OV14), 10 of the insertions were located in regions outside any predicted genes, four were located in exons, and one in an intron. Similarly, in *A. tumefaciens* (LBA4404 and EHA105) most of the events were located outside any predicted genes with one of them located in an exon and two located in a 3′ UTR region (Table 2).

**Table 2:** Genes and corresponding functions putatively disrupted by T-DNA insertion in via EMT and AMT

Sample	Gene	Intron/exon	Function
EMT			
11197/11199	LOC_Os07g48450.1	Intron	No apical meristem protein, putative, expressed
11280M1	LOC_Os06g51490.1	Exon	PHD-finger domain containing protein, putative, expressed
11470	LOC_Os10g02260.1	Exon	Peptide transporter PTR2, putative, expressed
11471	LOC_Os01g52380.1	Exon	Expressed protein
11694	LOC_Os02g13530.1	Exon	40S ribosomal protein S24, putative, expressed
AMT			
11379	LOC_Os04g43270.1	3′ UTR	WAX2, putative, expressed
11420	LOC_Os03g63540.1	3′UTR	Lysine-rich arabinogalactan protein 19 precursor, putative, expressed
11814	LOC_Os04g34440.1	Exon	Ubiquitin interaction motif-containing protein, putative, expressed

## Discussion

*A. tumefaciens* remains the primary bacterial species used for engineering novel crop varieties. Advantageous from the perspective of possessing a wide host range, freedom-to-operate restrictions still remain for the use of AMT be they region specific IP issues and/or the excessive cost of regulatory approval. Previously, Broothaerts et al. (2005) showed that three non-*Agrobacterium* species—*Rhizobium* sp. NGR234, *Sinorhizobium meliloti* and *Mesorhizobium loti*—were capable of genetically transforming different plant species. Using *S. meliloti*, Broothaerts et al. infected four of 687 rice calli (cv. Millin) obtaining an infection efficiency of 0.6 %. Meanwhile, for the same rice variety, infection with *A. tumefaciens* (EHA105) ranged from 50 to 80 %. In 2010, Rahmawati et al. assessed the effectiveness of *Rhizobium leguminosarum* and *S. meliloti* transformation compared to that of *A. tumefaciens*. Infecting Nipponbare (japonica), Rojolele (javanica) and Ciherang (indica) rice varieties, they obtained, GUS transient expression levels of 54, 63 and 46 % respectively with *A. tumefaciens* (LBA288-pCAMBIA 5106); 91, 71 and 68 % with *R. leguminosarum* (ANU845); and 0, 0.91 and 0 % with *S. meliloti*.

Recently Wendt et al. (2012) used *E. adhaerens* (OV14) for the production of *Phytophthora infestans*-resistant potato, obtaining an average shoot formation of 35 % in three different experiments. In the present study, we successfully established a reproducible protocol of calli transformation from Nipponbare, Curinga and IR64 rice varieties with the novel non-*Agrobacterium* species, *E. adhaerens* (OV14), with an infection efficiency of 90–100 %, 50–70 % and 80–100 %, respectively. These efficiencies are higher than previously reported for other non-*Agrobacterium* species (Broothaerts et al. 2005) and the corresponding transformation efficiency of 16.3 % for Curinga was in line with the relative efficiency of EMT with respect to AMT as previously reported in dicot crops (Wendt et al. 2012). In regards to Nipponbare, the transformation efficiency of 7.2 % indicates the protocol requires further modification to address escapes and hence obtain higher transformation efficiencies. During this study a single IR64 line was generated from 100 treated immature embryos which were used as the starting material (transformation efficiency: 1 %). Although low compared to our Japonica rice results, this transformation efficiency is high compared to other non-*Agrobacterium* species used for indica transformation (Rahmawati et al. 2010), where the rice variety Ciherang achieved 0.23 % of transformation efficiency.

In addition to examining the T-DNA transfer potential of *E. adhaerens* (OV14) in different rice varieties, this study also investigated the T-DNA integration patterns within the transgenic lines obtained. In the analysis of the flanking sequences none of the lines possessed erroneous vector backbone sequence. For the right border, it is important to note that the same integration patterns were obtained for *E. adhaerens* (OV14) and *A. tumefaciens* (both strains). When transforming different plant species with *A. tumefaciens*, several studies have reported finding 1–3 bases in the right border sequence: Zhai et al. (2004) using Chinese rice varieties (Minghui63, Yanhui559, Zhenxian97B, Peiai64, C418, Taihujing6, 8706, and Zhonghua11); Kim et al. (2003) in rice cv. Dongjin; Gheysen et al. (1990) using *A. thaliana*; Tinland (1996) with *A. thaliana* and tobacco; Kumar and Fladung (2002) using aspen; and Stahl et al. (2002) using barley. In our results, the junction point in the right border never went beyond these three bases regardless of whether the transformation occurred with *A. tumefaciens* or *E. adhaerens* (OV14). This site is a known cleavage site for generating single-stranded T-DNA fragments (Stachel et al. 1987; Yanofsky et al. 1986) and a pattern of integration where the T-DNA is partially deleted in the right or left borders can be explained by a model proposed by Gheysen et al. (1990).

In addition, the well-conserved right T-DNA end supports the hypothesis that the Vir D2 protein attached to the 5′ end accompanies or even guides the T-DNA to the plant nucleus, protecting the right border from degradation (Herrera-Estrella et al. 1988, 1990). Another pattern of insertion found in both borders for *A. tumefaciens* and *E. adhaerens* generated lines was the finding of filler sequences, which were found adjacent to the T-DNA that do not belong to adjacent rice sequence or vector sequence. In sample 11,470 a filler sequence of 59 bp was found, which, according to the BLAST analysis, is located on rice chromosome 5. Also we found at the right border of sample 11,253 a 25-bp insertion of a filler sequence from the nucleotides 5468–5495 from the T-DNA in the left border. The last finding can also be explained by one of Gheysen’s models denominating Complex Gap Repair. In some instances, the origin of the filler sequences found could not be determined because they were too short (6–8 bp) with homology to several places along the T-DNA and/or adjacent rice genomic DNA.

In addition, deletions of the genomic rice DNA were found in the site of T-DNA insertion in six EMT and 5 AMT lines evaluated. According to different models of T-DNA insertions, deletions occur when illegitimate recombination between the T-DNA and the genomic DNA takes place (Tinland 1996). These deletions have been reported previously in aspen plants (Kumar and Fladung 2002) and in *A. thaliana* (Forsbach et al. 2003; Mayerhofer et al. 1991); hence, these results suggest that T-DNA integration mechanisms are similar in monocotyledonous and dicotyledonous species and between *E. adhaerens* (OV14) and *A. tumefaciens*. Although mapping of T-DNA insertions from 22 independent transgenic lines did not reveal a significant bias of T-DNA insertion across the rice genome, there was a tendency for EMT delivered T-DNA to be inserted outside of gene coding regions of the genome, which would assist future regulatory evaluations of those lines engineered through EMT.

This paper confirms the propensity for EMT to genetically transform rice. While the original OV14 strain was isolated from the rhizosphere of a dicotyledonous plant, its utility to transform a monocot is significant. For EMT, the recorded T-DNA integration patterns were typical of illegitimate recombination models and were similar to that observed with AMT derived transgenic lines. Comparing the transformation efficiencies and integration patterns of both bacteria, we concluded that *E. adhaerens* (OV14) is indeed a promising and reliable non-*Agrobacterium* species with an expanding potential in crop biotechnology.

## Conclusions

Our results indicate that the non-plant pathogenic *E. adhaerens* (OV14) is able to transform embryogenic rice callus allowing the regeneration of transgenic plants. The integration patterns found in *E. adhaerens* (OV14) were similar to those found in *A. tumefaciens*. These analyses lead us to consider that *Ensifer*-mediated transformation is a reliable alternative to *Agrobacterium*–mediated transformation for researchers who work on the genetic transformation of rice.

## Materials and methods

### Plant material and tissue culture

Two japonica rice varieties (*Oryza sativa* L. spp. Curinga and Nipponbare) were selected for transformation with scutellar embryogenic calli used as target tissue. For this purpose, healthy mature dry seeds were manually dehusked, and surface sterilized by being hand shaken in a 50 % Clorox^®^ solution and a drop of Tween 20 for 10 min. Seeds were then rinsed with sterile distilled water 6–7 times before mature embryos (ME) were isolated under a stereoscope onto sterile moistened Whatman^®^ filter paper. For callus induction, 11 ME were placed scutellum side up on calli induction media (CHU-Ind, Table 3) (Chu 1978) and cultured for 20–25 days, after which the formed embryogenic calli were sub-cultured for 8–10 days in fresh CHU-Ind media in darkness at 24–26 °C to increase the callus size. Proliferated calli were then pre-cultured for 3 days on CHU-Ind-AS media supplemented with 100 µM of acetosyringone prior to transformation (in darkness at 24–26 °C). For the indica species (IR64) transformation, the seeds were dehusked under stereoscope and were surface sterilized by being hand shaken in 14 % Clorox^®^ solution and a drop of Tween 20 for 10 min. Seeds were rinsed with sterile distilled water 6–7 times. Isolation of immature embryos (IME) was carried out under stereoscope on sterile moistened Whatman^®^ filter paper. For IR64, 15–20 IME were directly isolated in plates supplemented with 300 µM of acetosyringone for subsequent transformation.

**Table 3:** Media composition for the transformation and regeneration of the japonica rice varieties Curinga and Nipponbare and the indica rice variety IR-64

Medium code	Use	Composition
CHU-Ind	Induction and proliferation of calli.	N6 salts and vitamins (Chu 1978), 100 mg l^−1^ Myo-inositol, 2.5 mg l^−1^ 2,4-d, 300 mg l^−1^ Casein enzymatic hydrolysate, Maltose 30 g l^−1^, Gelrite 3 g l^−1^. Autoclave and supplement l-proline 500 mg l mg l^−1^ and l-glutamine 500 mg l^−1^. pH 5.8
CHU-Ind-AS	Co-cultivation	Chu-Ind medium supplemented with 100 µM acetosyringone. pH 5.8.
CHU-infection	Agro-Infection	N6 salts and vitamins (Chu 1978), 100 mg l^−1^ Myo-inositol, 2,5 mg l^−1^ 2,4-d, 1 g l^−1^ Casamino acids, Maltose 15 g l^−1^, Glucose 15 g l^−1^. pH: 5.2. Filter sterilize. Add sterile acetosyringone 100 µM
CHU-H20 CHU-H40 CHU-H30	Selection	Chu-Ind medium supplemented with l-proline 500 mg l^−1^, l-glutamine 500 mg l^−1^, Gelrite 3 g l^−1^, 20 mg l^−1^ Hygromycin (*for Curinga*), 40 mg l^−1^ Hygromycin (for *Nipponbare*), 30 mg l^−1^ Hygromycin (for *IR64*), 250 mg l^−1^ Cefotaxime. pH 5.8
MS-H20 MS-H40 MS-H30	Shoot induction/regeneration	MS salts and vitamins (Murashinge and Skoog 1962), 100 mg l^−1^ Myo-inositol, 1 mg l^−1^ NAA, 30 g l^−1^ Sucrose, 3 g l^−1^ Gelrite. Autoclave and supplement with 4 mg l^−1^ Kinetin, 20 mg l^−1^ Hygromycin (for *Curinga*), 40 mg l^−1^ Hygromycin (for *Nipponbare*), 30 mg l^−1^ Hygromycin (*for IR64*), and 250 mg l^−1^ Cefotaxime. pH 5.8
MS-R-20 MS-R-40 MS-R-30	Rooting	MS salts and vitamins. 30 g l^−1^ Sucrose, 3 g l^−1^ Gelrite. Autoclave and supplement with, 20 mg l^−1^ Hygromycin (for *Curinga*), 40 mg l^−1^ Hygromycin (for *Nipponbare*), 30 mg l^−1^ Hygromycin (for *IR64*), 250 mg l^−1^ Cefotaxime. pH 5.8

### Bacterial strains used for transformation

Two *A. tumefaciens* strains, LBA4404 and EHA105, harbouring the plasmid pCAMBIA 1305.2, and *E. adhaerens* (OV14), equipped with the unitary plasmid pCAMBIA 5105 were used for rice transformation. pCAMBIA 5105 relies on spectinomycin and *npt II* (*neomycin phosphotransferase*) selectable marker genes for bacteria whereas pCAMBIA 1305.2 only relies on *npt II*. Both plasmids possess the *hpt II* (*hygromycin phosphotransferase*) gene as a plant selectable marker and both carry the *GUSPlus*-*His6* reporter gene.

### Transformation

A primary culture of each bacterial strain *A. tumefaciens* (LBA4404 and EHA105) and *E. adhaerens* (OV14) was prepared by inoculating a single colony from a freshly streaked YEP plate in 20 ml of liquid YEP medium. The *A. tumefaciens* cultures were supplemented with kanamycin (50 µg/ml^−1^) and rifampicin (60 µg/ml^−1^) and the *E. adhaerens* (OV14) with kanamycin (50 µg/ml^−1^) and spectinomycin (200 µg/ml^−1^). Each culture was incubated overnight in a rotatory shaker at 220–240 rpm in darkness at 28 °C for 16–18 h. After measuring the optical density of the cultures (*A. tumefaciens* = 0.5–0.6, and *E. adhaerens* = 0.3–0.5) the bacteria were centrifuged at 4000 rpm for 10 min and the supernatant discarded. The bacterial pellet was then re-suspended in 20 ml of CHU-infection media supplemented with 100 µM of acetosyringone. The resulting culture was then incubated at 200–220 rpm at 21 °C in darkness. For IR64 transformation using *E. adhaerens* it was necessary to set the O.D._600_ to 1.0. The bacterial culture was centrifuged at 4000 rpm for 10 min and the supernatant discarded. Then the bacterial pellet was re-suspended in 20 ml of CHU-infection media supplemented with 100 µM of acetosyringone. The resulting culture was then incubated in darkness at 200–220 rpm and 21 °C during 1.5–3 h, according to the literature, incubation at low temperature cooperates with the induction of the virulence genes (Baron et al. 2001; Dillen et al. 1997; Hiei et al. 1997).

### Tissue infection, selection and regeneration of plants

For tissue infection, the Curinga rice was infected with *A. tumefaciens* LBA4404, and the Nipponbare was infected using both *A. tumefaciens* strains, LBA4404 and EHA105. Pre-cultured embryogenic calli of Curinga and Nipponbare (20–30 calli per plate) each received a single drop (~5 µl) of the respective bacterial suspension. Co-cultivation of the tissue was for 3 days for both bacterial species infecting the japonica rice varieties and 5–7 days for *E. adhaerens* infecting IR64; in both cases the cultures were put in darkness at 21 °C. After co-cultivation, bacterial growth was prevented by washing with autoclaved distilled water plus cefotaxime (500 mg l^−1^ for the japonica species and 250 mg l^−1^ for the indica species), and the tissue was dried on sterile filter paper. At this step, tissue (calli and IME) were GUS tested (Jefferson et al. 1989) in order to evaluate the infection efficiency (IE) of both bacterial species with % IE calculated as the number of calli containing blue spots × 100/total number of calli treated.

Following co-cultivation, explants were transferred to selection media amended with hygromycin. For Nipponbare and Curinga, previous assays determined a minimum inhibitory concentration of 40 mg l^−1^ and 20 mg l^−1^ of hygromycin, respectively (data not shown). Selection proceeded for 20–30 days at 24–26 °C in the dark and after the first round of selection, brown or black calli were removed and only white-creamy embryogenic calli were sub-cultured to fresh CHU-H20 medium for a further 15–20 days. After this selection, actively growing pieces of calli were transferred to shoot induction medium (MS-20H for Curinga; MS-40H for Nipponbare) containing hygromycin (20–40 mg l^−1^) plus cefotaxime (250 mg l^−1^) for incubation at 24–26 °C under a 16-h photoperiod until vegetation tissue was visible (in some cases, it was necessary to refresh the media for some calli between days 20 and 30). Following shoot induction, approximately 2–3 cm plantlets were transferred to rooting media (MS-R for Curinga; MS-R for Nipponbare) supplemented with hygromycin (20–40 mg l^−1^) and cefotaxime (250 mg l^−1^). For IR64, plant media set and culture regimes were the same as for the japonica varieties, but selection was performed using hygromycin 30 mg l^−1^ (Advanced Indica Rice Transformation Training, IRRI, 2012). Transformation efficiency (TE) was calculated as the number of Southern blot positive plants × 100/number of infected calli.

### Molecular characterization of putative transformants

For hardening purposes, plantlets 7–10 cm long were transferred to falcon tubes or glass jars and covered with a small translucent bag to maintain humidity for 3–4 days at 24–26 °C with a 16-h photoperiod, after which plantlets were transferred to a hydroponic solution protocol (Subbarao et al. 2007). Within 10 days, a 5–7 cm long (100–150 mg) section of leaf tissue was excised for DNA extraction using an Alkyltrimethylammonium bromide (MATAB) modified method (Murray and Thompson 1980) and chloroform:isoamylalcohol (24:1). PCR analysis was used to confirm the presence or absence of the transgene in primary transformants (T_0_) with the constitutive gene *OsNAC* and the selection gene *hpt II* employed to assess DNA quality and transgene identification, respectively. The primer sequences were: OsNAC6-Rv 5′-GTTACTCG TGCATGATCCAC-3′, OsNAC6/n5′-ATGAGCGGCGGTCAGGACCTGCA-3′ (Acc. No EU84 6993.1), HPT-RKFw5′C TATTCCTTTGCCTTCGGACG-3′ and HPT-RKRv 5′-CTCCGCATTGGTCTTGACCA-3′. In both genes, the 20 µl reaction mixture contained 1X GoTaq^®^ Green Master mix (Promega Cat. No. M7123), 0.25 µM of each primer and ~50 ng of template. Cycling conditions were 95 °C (2 min), followed by 35 cycles of denaturation at 94 °C (30 s), annealing at 55 °C (30 s) and extension at 72 °C (1 min), with a final extension of 72 °C (5 min). Five µl of the PCR product were resolved in a SYBR-Safe 1 % stained gel. To verify the copy number of the transgenic plants, Southern blot was performed by digesting 10 µg of DNA using 50 U of the enzyme EcoRI (Life technologies ^®^) Additional file 2: Figure S1. Digested DNA was resolved in a 1 % agarose gel and transferred on a nitrocellulose membrane (GE Healthcare) before probing a 228 bp fragment of the *hpt II* gene chemically labelled with Dig-dUTP (ROCHE^®^). Hybridization was performed at 42 °C overnight and the membrane washing was completed according to the manufacturer’s instructions. Transgene integration analysis was conducted on single copy lines as confirmed via Southern blotting. An adapter ligation PCR approach from Hagiwara and Harris (1996) was used to extract the flanking chromosomal rice sequences from the T-DNA in the left border of both plasmids, pCAMBIA 5105 and pCAMBIA 1305.2. DNA was digested using the blunt-end cutters, AfeI, MscI and SfoI.

At the same time, the following oligonucleotides, V-top 5′-GAAGGAGAGGACGCTGTCTGT CGAAGGTAAGGAACGGACGAGAGAAGGGAGAG-3′ and V-bottom 5′CTCTCCCTTCT C GAATCGTAACCGTTCGTACGAGAATCGCTGTCCTCTCCTTC-3′, were synthesized, purified and dissolved in distilled water to a final concentration of 4 µM each to form the “vectorette unit”, which was linked to the digested DNA. V-top and V-bottom sequences are complementary to each other except in the middle part, where the primer, 224M13 (5′-TGTAAAACGACGGCCAGTCGAATCGTAACCGTTCGTACGAGAATCGCT-3′), attaches to the bubble-like structure formed allowing the amplification of the unknown DNA rice region and respective adjacent vector sequence. The sequences obtained for the right border were analysed using the BLAST tool from the Rice Genome database (http://rice.plantbiology.msu.edu/-Os-Nipponbare-Reference-IRGSP-1.0 Pseudomolecules) to obtain the position, flanking sequences and T-DNA integration pattern of the sequences. Using the data retrieved from BLAST, we designed primers within the rice genomic sequence adjacent to the T-DNA insertion in the left border to amplify and isolate the flanking sequence.

### Growth conditions for T_0_ plants and T_1_ seed production

PCR-positive T_0_ plants from the three varieties were moved into hydroponic cultures in the greenhouse and grown according to Subbarao et al. (2007) for 2 weeks. Once Southern blot analyses were completed, T_0_ plants with single inserts and well-established root systems were subsequently transplanted into clay soil for further growth. Self-pollinated progenies (T_1_ seeds) were harvested within a 3-month period. The T_1_ seeds were manually harvested, cleaned and stored at 4 °C.

## Additional files

10.1186/s40064-015-1369-9 **Rice DNA/T-DNA border sequences.** T-DNA REF: corresponds to the native T-DNA sequence plus the complete right border. **blue:** fragments of the right border sequences; **orange:** fragments of the left border sequences; **green:** adjacent rice genomic DNA sequence; **red:** filler sequences; **underlined:** microhomologies; **capital black letter:** T-DNA fragment.
10.1186/s40064-015-1369-9 Schematic representation of the plasmid pCAMBIA 5105 indicating EcoRI restriction site and luciferase probe used for Southern blot analysis.
