# Supplementary material for: Ensifer-mediated transformation: an efficient non-Agrobacterium protocol for the genetic modification of rice
Source: Springerplus. 2015 Oct 13;4:600. doi: 10.1186/s40064-015-1369-9 (PMC4628045; doi:10.1186/s40064-015-1369-9)
Supplement: Supplementary file 1 — 10.1186/s40064-015-1369-9 Rice DNA/T-DNA border sequences. T-DNA REF: corresponds to the native T-DNA sequence plus the complete right border. blue: fragments of the right border sequences; orange: fragments of the left border sequences; green: adjacent rice genomic DNA sequence; red: filler sequences; underlined: microhomologies; capital black letter: T-DNA fragment. [file 40064_2015_1369_MOESM1_ESM.doc]

**Additional file 1: Table S1.** **Rice DNA/T-DNA border sequences.** T-DNA REF: corresponds to the native T-DNA sequence plus the complete right border. **blue:** fragments of the right border sequences; **orange:** fragments of the left border sequences; **green:** adjacent rice genomic DNA sequence; **red:** filler sequences; **underlined:** microhomologies; **capital black letter:** T-DNA fragment.

RIGHT BORDER

| **SAMPLE ID** | **SEQUENCE** | **Strain/**  **species** | **Var.** |
| --- | --- | --- | --- |
| **TDNA REF** | CAAATGGGCGGTTATATAGGACAGTTTGTGACTATCAAATTAAGGGCTAGATCATTGTATCTACTGTGGCGCGCGCT |  |  |
| 11232 | **TCACATGTTGTTACTCGTCGGG**AGTTTGTGACTATCAAATTAAGGGCTAGATCATTGTATCTACTGTGGCGCGCGCT | L | C |
| 11379 | **CAAAAAGGAAGGAAAATCCCGAGC**TTTTGACTATCAAA-TTAAGGGCTAGATCATTGTATCTACTGTGGCGCGCGCT | L | C |
| 11291 | **CAGATGGATCTCTCTAAAACCTAA**TTTGTGACTATCAAATTAAGGGCTAGATCATTGTATCTACTGTGGCGCGCGCT | L | C |
| 11289 | **TCTACCTGCCGCCTCGGGAGTTCGGGACTTACCTCGG**AATTAAGGGCTAGATCATTGTATCTACTGTGGCGCGCGCT | L | C |
| 11309 | **CATGCCTGCCTCCACTACGAGGTGTGCAACGGCGGTAACTAG**-----177 BP TDNA LOST-----**TGCA**GTACG | L | C |
| 11420 | **GTCATATAGTAAGATGATCCATATGTTTTGACGCACATAGGT**-----177 BP TDNA LOST-----TGCAGTACG | L | N |
| 11422 | **CCACCGGCGAGAAGCAAAA**GCAAGTTTGTGACTATCAAATTAAGAGCTAGATCATTGTATCTACTGTGGCGCGCGCT | L | N |
| 11645 | **CTTGCGGTTGCTCTCCTTCTT**GTACTGGTGATTATCAAATTAAGGGCTAGATCATTGTATCTACTGTGGCGCGCGCT | EH | C |
| 11814 | **GTGGAGGAAGAGTTCGAACAACGGC**TTGTGACTATCAAATTAAGGGCTAGATCATTGTATCTACTGTGGCGCGCGCT | EH | C |
| 11197/99 | **TTGACGTATAGGTAGGTAGAGG**AGTTTGTGACTATCAAATTAAGGGCTAGATCATTGTATCTACTGTGGCGCGCGCT | Ens | N |
| 11470 | **TCGAATGAAGTTGTGCA**GCGGGAGCCAACGTACTGACTGTCTACCTGCCGCCTCGGGAGTTCGGGACTTACCTCGGA | Ens | N |
| 11418 | **TCCTAGACTAATA**TGCA*CCATG*AGTTTGTGACTATCAAATTAAGGGCTAGATCATTGTATCTACTGTGGCGCGCGCT | Ens | N |
| 11436 | **TATTTGACACAAAAATGATTATTTC**TAGATCATATCAAATTAAGGGCCAGATCATTGTATCTACTGTGGCGCGCGCT | Ens | N |
| 11473 | **AGGTATATTATAAGACAACCGA**AGTTTGTGACTATCAAATTAAGGGCTAGATCATTGTATCTACTGTGGCGCGCGCT | Ens | C |
| 11267 | **TCCGTTCTCACTACGA**TGCATGCGTTTGTGACTATCAAATTAAGGGCTAGATCATTGTATCTACTGTGGCGCGCGCT | Ens | C |
| 11414 | **GTCCGCGACGAGGAGGCACTCTGA**TTTGTGACTATCAAATTAAGGGCTAGATCATTGTATCTACTGTGGCGCGCGCT | Ens | C |
| 11471 | **CTTCTTCTATAACGTGTCGGAATCTC**TGTGACTATCAAATTAAGGGCTAGATCATTGTATCTACTGTGGCGCGCGCT | Ens | C |
| 11253 | **TCACCGCACC**TAAAATCATGACCTAAAACCAAAATCAAATTAAGGGCTAGATCATTGTATCTACTGTGGCGCGCGCT | Ens | C |
| 11260 | **CTTCTACCACTAAAGAAAAAGATAG**TTGTGACTATCAAATTAAGGGCTAGATCATTGTATCTACTGTGGCGCGCGCT | Ens | C |
| 11694 | **GCCTAGGCGGACGTGTGTGCGTGTGCGTGTGC**TATCAAATTAAGGGCTAGATCATTGTATCTACTGTGGCGCGCGCT | Ens | C |
| 11813 | **ACGTGACGCGCCGCTTGTGGCCGTG**TTGTGACTATCAAATTAAGGGCTAGATCATTGTATCTACTGTGGCGCGCGCT | Ens | C |
| 11280 M1 | **TACCCAGTCGTTGTCATCATCGGTTACCCTCTTTTGTGTACATTCCC**-----200 BP TDNA LOST-----ATGC | Ens | C |

| **SAMPLE ID** | **SEQUENCE** | **Strain** | **Var.** | **Deleted genomic DNA** |
| --- | --- | --- | --- | --- |
| **TDNA REF** | **ACCGTCCTATATAACACCACATTTGTTTAACTGCGAATCTGTTGAATTATTGTGTAACGCCTGCAAAAATTACA** |  |  |  |
| 11232 | **ACATACCGAT**ATAACACCACATTTGTTTAACTGCGAATCTGTTGAATTATTGTGTAACGCCTGCAAAAATTACA | L | C | 14 |
| 11379 | **GGAATGAC**ATATAACACCACATTTGTTTAACTGCGAATCTGTTGAATTATTGTGTAACGCCTGCAAAAATTACA | L | C | 25 |
| 11291 | **TAAAAGCAGGCATCAG**CCACATTTGTTTAACTGCGAATCTGTTGAATTATTGTGTAACGCCTGCAAAAATTACA | L | C | 84 |
| 11645 | **CACGCAAAGACGAATCTCCGGGCACATTAATTG**-----100 bp TDNA lost----CCCTAGACCTAAAATC | EH | C | 176 |
| 11422 | **TCTTCTTTTCTTTTCTTCTCTTTTGCGTCTTCTC**TTTTGGTTGAGATAGT--277 bp TDNA lost--CTCG | L | N | 11 |
| 11197/99 | **TCAAGTTAATCAT**CACCACG**T**TTTGTTTAACTGCGAATCTGTTGAATTATTGTGTAACGCCTGCAAAAATTACA | ENS | N | 37 |
| 11260 | **CTGGTA**CTATATAACACCACATTTGTTTAACTGCGAATCTGTTGAATTATTGTGTAACGCCTGCGAAAATTACA | ENS | C | 50 |
| 11473 | **GCGAAT**CTATATAACACCACATTTGTTTAACTGCGAATCTGTTGAATTATTGTGTAACGCCTGCAAAAATTACA | ENS | C | 273 |
| 11471 | **ATAATACTAGAA**--31 bp TDNA lost--TGCGAATCTGTTGAATTATTGTGTAACGCCTGCAAAAATTACA | ENS | C | 23 |
| 11694 | **AGGCCACTGGCAGGCCCGA**ATCTAGTTTAACTGCGAATCTGTTGAATTATTGTGTAACGCCTGCAAAAATTACA | ENS | C | 35 |
| 11813 | **GCACGTCCTCCACCG**ACCACATTTGTTTAACTGCGAATCTGTTGAATTATTGTGTAACGCCTGCAAAAATTACA | ENS | C | 16 |

LEFT BORDER
