# Supplementary material for: Ensifer-mediated transformation: an efficient non-Agrobacterium protocol for the genetic modification of rice
Source: Springerplus. 2015 Oct 13;4:600. doi: 10.1186/s40064-015-1369-9 (PMC4628045; doi:10.1186/s40064-015-1369-9)
Supplement: Supplementary file 2 — 10.1186/s40064-015-1369-9 Schematic representation of the plasmid pCAMBIA 5105 indicating EcoRI restriction site and luciferase probe used for Southern blot analysis. [file 40064_2015_1369_MOESM2_ESM.doc]

Additional files

**Additional file 2: Figure S1.** Schematic representation of the plasmid pCAMBIA 5105 indicating EcoRI restriction site and luciferase probe used for Southern blot analysis.
